# Tuberculosis Treated by Marmorek's Serum

**Published:** 1905-11-11

**Authors:** 


### TUBERCULOSIS TREATED BY MARMOREK'S SERUM.

Dr. Harold F. Bassano [1] has used Dr. Marmorek's antituberculous serum in five cases extending over a period of sixteen months, and considers that the results warrant further trial of the remedy. He indicates the following conclusions: (1) That the beneficial effects are most conspicuous in the surgical forms of tuberculosis. (2) That, except for occasional slight general malaise, local urticarial rash, and pain at the point of injection, no objectionable results follow the use of the serum. (3) That after injection pyrexia is diminished though this effect may be preceded by a slight initial rise of temperature. (4) That in cases of tuberculous peritonitis and in hip-joint disease pain is much relieved. (5) That in phthisis pulmonalis the amount of sputum is lessened after, it may be, some temporary increase of the expectoration. (6) That with the arrested progress of the disease the general health rapidly improves, and the patient gains in weight. Regarding the dose and method of administration, Dr. Marmorek advises the injection of five cubic centimetres every other day for three weeks, this to be followed by a three weeks interval, when the injections are repeated. In one of his cases Dr. Bassano injected sixty cubic centimetres in the course of twelve days, and then allowed a month's interval, this being attended with good results. The site of injection should be either the abdominal wall or the thighs, and the needle should be introduced as deeply as possible without penetrating the deep fascia. An all-glass syringe should be used, and strict antiseptic precautions observed.

[1] *Lancet*, September 9, 1905.

